# Antinociceptive Effects of Analgesic-Antitumor Peptide (AGAP), a Neurotoxin from the Scorpion *Buthus martensii* Karsch, on Formalin-Induced Inflammatory Pain through a Mitogen-Activated Protein Kinases–Dependent Mechanism in Mice

**DOI:** 10.1371/journal.pone.0078239

**Published:** 2013-11-14

**Authors:** Qinghong Mao, Jiaping Ruan, Xueting Cai, Wuguang Lu, Juan Ye, Jie Yang, Yang Yang, Xiaoyan Sun, Junli Cao, Peng Cao

**Affiliations:** 1 Jiangsu Branch of China Academy of Chinese Medical Sciences, Nanjing, China; 2 Laboratory of Cellular and Molecular Biology, Jiangsu Province Institute of Chinese Medicine, Nanjing, China; 3 Jiangsu Key Laboratory of Anesthesiology, Department of Anesthesiology, Affiliated Hospital of Xuzhou Medical College, Xuzhou, China; Institute Pasteur, France

## Abstract

In the present study, we investigated the anti-nociceptive effect and the underlying mechanism of the analgesic-antitumor peptide (AGAP), a neurotoxin from the scorpion Buthus martensii Karsch. AGAP in doses of 0.2, 1 and 5 µg was injected intraplantarly (i.pl.) before formalin injection 10 min at the same site. The suppression by intraplantar injection of AGAP on formalin-induced spontaneous nociceptive behaviors was investigated. The results show that AGAP could dose-dependently inhibit formalin-induced two-phase spontaneous flinching response. To investigate the mechanism of action of treatment with AGAP in inflammatory pain, the expressions of peripheral and spinal phosphorylated mitogen-activated protein kinases (phospho-MAPKs) including p-p38, p-ERK and p-JNK were examined. We found that formalin increased the expressions of peripheral and spinal MAPKs, which were prevented by pre-intraplantar injection of AGAP in inflammation pain model in mice. AGAP could also decrease the expression of spinal Fos induced by formalin. Furthermore, combinations the lower doses of the inhibitors of MAPKs (U0126, SP600125, or SB203580 0.1 µg) with the lower dose of AGAP (0.2 µg), the results suggested that AGAP could potentiate the effects of the inhibitors of MAPKs on the inflammatory pain. The present results indicate that pre-intraplantar injection of AGAP prevents the inflammatory pain induced by formalin through a MAPKs-mediated mechanism in mice.

## Introduction

Scorpion Buthus martensii Karsch (BmK) has been one of the indispensable materials used as traditional Chinese medicine in the treatment of convulsions and epilepsy since the Sung Dynasty [1]. Scorpion toxin contains various toxic polypeptides with different functions. Moreover, these polypeptides have been shown to affect the activities and functions of ion channels, such as sodium and potassium channels [2], [3]. Antitumor-analgesic peptide (AGAP), one of these polypeptides, has been purified from the venom of Mesobuthus martensii Karsch, which is widely distributed in China [4]. Studies have shown that AGAP has analgesic and antitumor activities [4], [5].

The formalin test is commonly employed as a model of acute and tonic pain. Formalin injection in mice can produce two phases of nociceptive behaviors. The first, or acute, phase lasts for about 5 min. After a short quiescent period, the second, or tonic, phase is triggered for 15–30 min [6]. Liu et al. reported that intrathecal injection of BmK could dose-dependently decrease formalin-induced spontaneous nociceptive behaviors and spinal c-Fos expression in rats [6].

MAPKs, including p38, ERK, and JNK, are a family of serine/threonine protein kinases that transduce extracellular stimuli into intracellular posttranslational and transcriptional responses. It is well established that the mitogen-activated protein kinases (MAPKs) activation is involved in the modulation of nociceptive information and peripheral and central sensitization produced by intense noxious stimuli [7]–[12]. Our previous study showed that AGAP down-regulated the protein expression of p-p38, p-JNK, and p-Erk1/2 in *vitro* [13]. However, the effect and mechanism of AGAP on acute inflammation pain in *vivo* is still unknown.

In the current study, our results showed that AGAP dose-dependently and significantly reduced paw elevation and paw licking time, during both the early and late phases. Formalin-induced inflammatory pain was accompanied with activation of peripheral and spinal MAPKs, and also was prevented by pre-treatment with AGAP.

## Materials and Methods

## Animals

Adult, male Kunming mice (20–25 g) were employed in these present studies. Mice were housed under a 12 h/12 h light–dark cycle regime, with free access to food and water. The animals were provided by Experimental Animal Center of Jiangsu Province Institute of Traditional Chinese Medicine. All experimental protocols were approved by the Animal Care and Use Committee of Jiangsu Branch of China Academy of Chinese Medical Sciences and were in accordance with the Declaration of the National Institutes of Health Guide for Care and Use of Laboratory Animals (Publication No. 80-23, revised 1996).

### Drug application

Drugs or vehicles were administered in a volume of 10 µL into the plantar surface of the right hind paw using a 25 µL Hamilton syringe with a 28 gauge needle. The needle was inserted into the plantar skin proximal to the midpoint of the hind paw and advanced about 10 mm so that it reached the midpoint of the hind paw, where the solution was injected forming a bleb that disappeared within 10 min.

All doses of drugs are based on the results of preliminary experiments. The doses of each drug and time points of treatment were presented in the parts of Section 3 and figure legends.

### The preparation for AGAP

AGAP was obtained by the expression of pET28a/SUMO-AGAP in *Escherichia coli* as described [5]. The activity of AGAP was the same as the previous and dissolved in saline.

### Formalin test

The procedure used was essentially the same as that reported by Hunskaar and Hole [14]. Approximately 30 min before testing, mice were individually placed in Perspex observation chambers (10×20×15 cm) for adaptation. Then, the animals were taken out of the chamber, and 10 µL of 2% formalin in 0.9% saline was injected subcutaneously into the dorsal surface of the right hind paw with a 25 µL Hamilton syringe with a 28 gauge needle. Immediately after formalin injection, each mouse was returned to the observation chamber. The amount of time spent licking and biting the injected paw were measured from 0 to 5 min (the first phase) and from 10 to 40 min (the second phase) after formalin injection, and was considered as indicative of nociception.

### Immunohistochemistry

Mice were anesthetized with sodium pentobarbital (60 mg/kg i.p.) and underwent sternotomy, intracardially perfused with 20 mL saline followed by 100 mL of 4% ice cold paraformaldehyde in 0.1 mol/L phosphate buffer (PB). The skin of injective site and spinal cord of L4–5 were removed, post-fixed in 4% paraformaldehyde for 3 h, and subsequently allowed to equilibrate in 30% sucrose in PB overnight at 4°C. 10 µm transverse series sections were cut on a cryostat and stored in PB. After washing in phosphate buffer saline (PBS), the tissue sections were incubated in PBS containing 5% normal goat serum and 0.3% TritonX-100 at room temperature for 30 min, followed by primary polyclonal rabbit anti-pERK1/2 antibody (1∶200), primary polyclonal rabbit anti-p-p38 antibody (1∶200) or primary polyclonal rabbit anti-pJNK antibody (1∶200) for skin slices, or primary polyclonal rabbit anti-Fos antibody (1∶1000) for the spinal cord slices at 4°C for 48 h (all antibodies were obtained from Santa Cruz Biotechnology, CA, USA). The sections were then incubated in biotinylated goat anti-rabbit (1∶200) at 37°C for 1 h and in avidin-biotin-peroxidase complex (1∶100) (Vector Labs, CA, USA) at 37°C for 2 h. Finally, the sections were treated with 0.05% diam-inobenzidine (DAB) for 5–10 min. Sections were rinsed in PBS to stop the reaction, mounted on gelatin-coated slides, air-dried, dehydrated with 70–100% alcohol, cleared with xylene, and cover-slipped for microscopic examination.

For the analysis of the change of Fos protein expression, we examined five spinal cord sections per animal, selecting the sections with the greatest number of positive neurons. For each animal, we recorded the total number of positive neurons in the ipsilateral spinal cord. All positive neurons were counted without considering the intensity of staining.

### Western blot analysis

The spinal cord of L4–5 were quickly extracted and stored in liquid nitrogen. Tissue samples were homogenized in lysis buffer containing (in mM) Tris 20.0, sucrose 250.0, Na_3_VO_4_ 0.03, MgCl_2_ 2.0, EDTA 2.0, EGTA 2.0, phenylmethylsulfonyl fluoride (PMSF) 2.0, dithiothreitol (DTT) 1.0, protease inhibitor cocktail 0.02% (v/v), pH 7.4. The homogenates were centrifuged at 5000 g for 30 min at 4°C. The protein was denatured in boiled water for 5 min and centrifuged at 12,000 rpm for 5 min. Equal amounts of protein were separated on 8% or 13% SDS-PAGE by electrophoresis and blotted onto PVDF membrane using the SemiPhor Semi-Dry transfer unit. Membranes were blocked for 2 h in 1% BSA (in TBST) at room temperature and then immunobloted overnight at 4°C with primary antibodies. After three washes in 0.05% Tween-20 in TBS (TBST), membranes were incubated with goat anti-mouse IgG-HRP or goat anti-rabbit IgG-HRP second antibodies (Santa Cruz) for 2 h at room temperature. Membranes were washed three times in TBST. Immunoreactive protein bands were detected with Gel/Chemi Doc System (Bio-Rad). The grayscale of the bands was detected by Adobe Photoshop CS to obtain the semi-quantitative analysis.

### Statistical analysis

Changes in behavior and the changes of expression of p-MAPKs among groups were tested with two-way ANOVA with repeated measures followed by Bonferroni post hoc tests. Individual t-test was used to test the number of Fos expression about differences between the test group and the control group. All data are presented as means ± SD. Statistical results are considered significant if *P*<0.05.

## Results

### Pre-intraplantar injection of AGAP dose-dependently prevents the inflammatory pain induced by formalin

**Figure 1:**
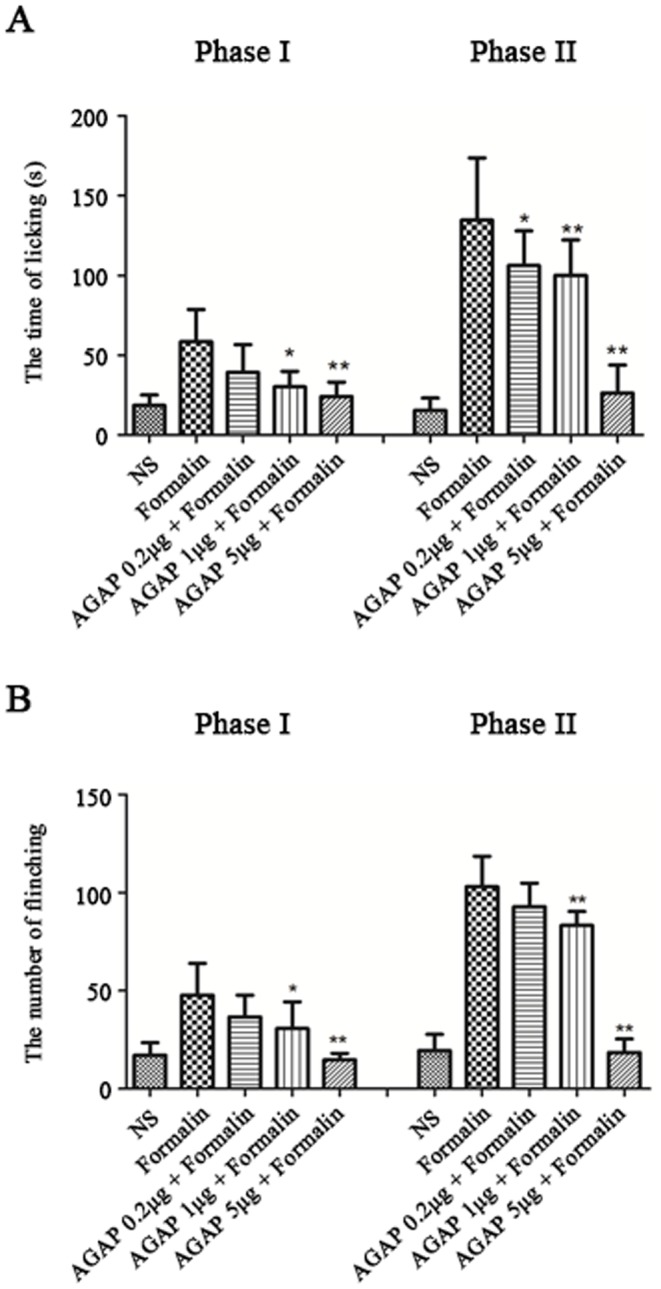
Pre-intraplantar injection AGAP prevents the inflammatory pain induced by formalin in a dose-dependent manner. Intraplantar injection of 10 µL of 2% formalin in 0.9% saline produced Spontaneous pain. AGAP (0.2 µg, 1 µg, 5 µg) were injected at 10 min before formalin injection. Spontaneous pain were recorded within 0∼5 min (Phase I) and 15∼30 min (Phase II) after formalin injection. Pre-treatment of AGAP decreased the time of licking/biting paw (A) and the number of flinching paw (B). *P<0.05, **P<0.01 compared with formalin group, n = 8 mice in each group.

Formalin injection in mice can produce two phases of nociceptive behaviors. The first phase (0–5 min) and the second phase (15–30 min). We want to know if pre-treatment AGAP could prevent the inflammatory pain induced by formalin. To address this question, various doses of AGAP (0.2, 1, 5 µg in 10 µL saline) were injected at 10 min before injection of formalin at the same site. The total time of paw licking and number of paw elevation in the saline-injected or AGAP injected control group was similar in phase I and phase II. Figure 1 shows the effects of pre-intraplantar AGAP during the study period. AGAP dose-dependently and significantly reduced paw elevation and paw licking time, during both the early and late phases.

### AGAP down-regulated the expressions of spinal p-MAPKs both in Phase I and II

**Figure 2:**
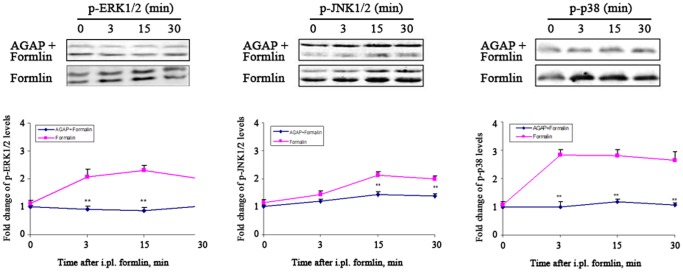
Pre-intraplantar injection AGAP prevents the expression of spinal MAPKs induced by formalin both in Phase I and Phase II. AGAP (5 µg) were injected at 10 min before formalin injection. The expression of spinal phospho-MAPKs was assayed at 0,3,15 and 30 min after formalin injection. The representative bands for the expressions of p-p38, p-ERK, and p-JNK in spinal after injection of formalin (Above). The quantitative data for the expression of each phospho-MAPKs (Below). The fold change of phospho-MAPKs levels in AGAP+Formalin group at 0 min time-point was set at 1 for quantifications. **P<0.01 compared with Formalin group, n = 4 mice in each group.

Some studies have shown that peripheral MAPKs activation mediated inflammatory or injury-induced peripheral sensitization and hyperalgesia [15], [16]. We also found that AGAP down-regulated the protein expression of p-MAPKs in vitro [13]. The behaviors results suggested that AGAP dose-dependently prevents the inflammatory pain induced by formalin in both Phase I and II. Thus, we tested the expressions of p-MAPKs at 0, 3, 15, 30 min time points after formalin injection by western-blot. The results showed the expressions of p-p38 and p-ERK were significantly decreased by pre-treatment with AGAP, while the expression of p-JNK was no deference from the control group in Phase I. The expressions of p-JNK p-ERK, p-p38 were significantly decreased by pre-treatment with AGAP in Phase II. (Fig. 2).

### Pre-intraplantar injection AGAP down-regulated the expressions of skin and spinal p-MAPKs induced by formalin

**Figure 3:**
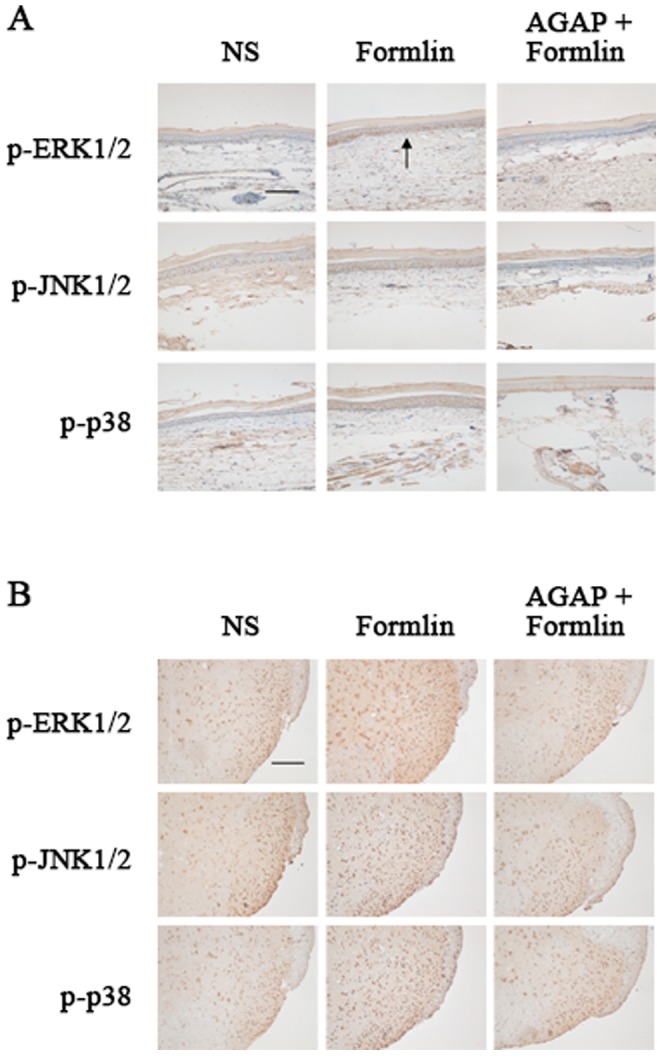
Pre-intraplantar injection AGAP prevents the expressions of skin and spinal MAPKs induced by formalin. AGAP (5 µg) were injected at 10 min before formalin injection. The expressions of skin and spinal phospho-MAPKs in immunohistochemical experiments were detected at 15 min time-point. (A) Representative immunohistochemical staining for the expression of p-p38, p-ERK1/2, and p-JNK1/2 in skin after injection of formalin, n = 6 mice in each group, Scale bar = 200 µm. (B) Representative immunohistochemical staining for the expression of p-p38, p-ERK, and p-JNK in spinal after injection of formalin, n = 6 mice in each group, Scale bar = 200 µm.

Furthermore, we tested whether AGAP prevents formalin-induced expressions of p-MAPKs at 15 min after formalin injection. The immunohistochemistrical results showed the expressions of skin and spinal p-JNK p-ERK and p-p38 were significantly decreased by pre-treatment with AGAP. We also found that formalin-induced p-MAPKs expression is predominately located in the skin and in the superficial layers of spinal dorsal horn (DH) neurons, which can be partially prevented by pre-intraplantar AGAP (Fig. 3A and B).

### AGAP down-regulated the expressions of spinal p-MAPKs induced by formalin in dose-dependent manner

**Figure 4:**
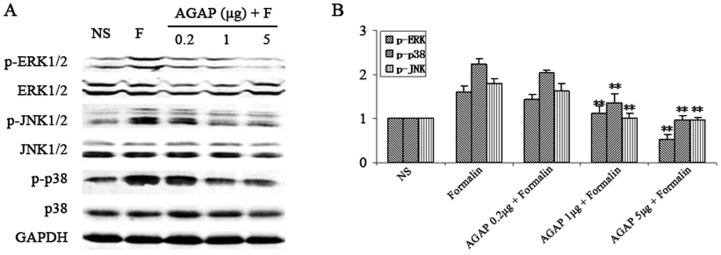
Pre-intraplantar injection AGAP prevents the expression of spinal MAPKs induced by formalin in a dose-dependent manner. AGAP (0.2 µg, 1 µg, 5 µg) were injected at 10 min before formalin injection. The expression of spinal MAPKs was assayed at 15 min after formalin injection. (A) The representative bands for the expressions of p-p38, p-ERK, and p-JNK in spinal after injection of formalin. (B) The quantitative data for the expression of each phospho-MAPKs. The fold change for the density of each phospho-MAPKs normalized to total MAPKs for each sample. The fold change of phospho-MAPKs levels in NS group was set at 1 for quantifications. *P<0.05, **P<0.01 compared with Formalin group, n = 4 mice in each group.

The behaviors results suggested that pre-treatment with AGAP dose-dependently prevent the total time of paw licking and number of paw elevation induced by formalin. The above western-blot data showed that the expressions of p-JNK p-ERK, p-p38 were significantly decreased by pre-treatment with AGAP in Phase II. According to the behaviors tests, the western-blot data showed that expressions of spinal p-ERK,p-p38 and p-JNK were decreased by pre-treatment with AGAP in a dose-dependent (AGAP 0.02, 0.1, 0.5 µg in 10 µL saline) manner (Fig. 4A and B) at 15 min after formalin injection.

### Pre-treatment with AGAP prevented the spinal Fos expression induced by formalin

**Figure 5:**
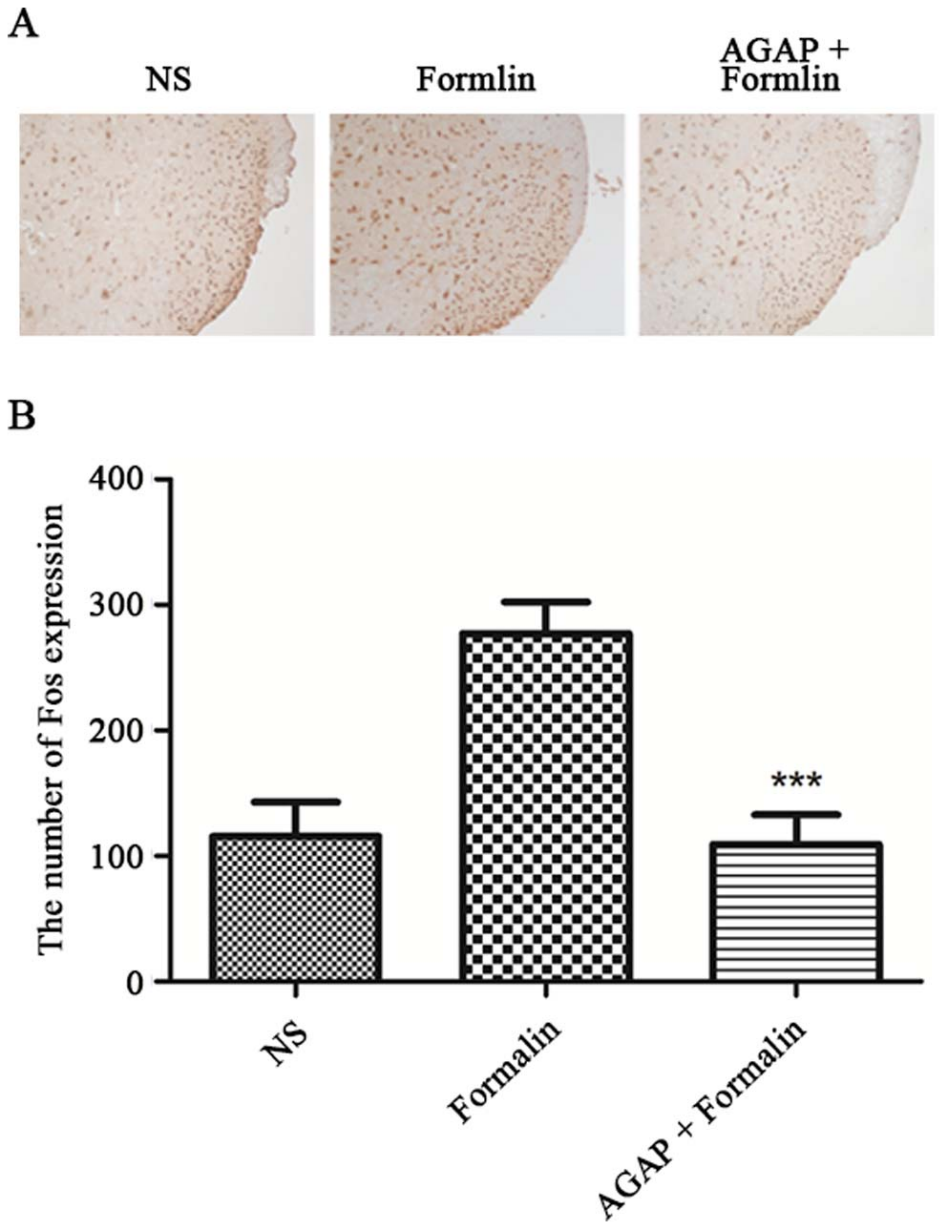
Pre-treatment with AGAP inhibited formalin-induced spinal Fos protein expression. AGAP (5-µg) was injected at 15 min before formalin injection. The expression of spinal Fos protein in immunohistochemical experiments was detected at 15 min after formalin injection. Representative immunohistochemical staining (A) and the quantitative data (B) for the decreased formalin-induced Fos expression by pre-treatment with AGAP (5 µg) not saline, in the spinal cord of mice. **P<0.01 compared with formalin group, n = 6 mice in each group, scale bar = 200 µm.

The expression of Fos protein also may be a useful tool to examine the effectiveness of different analgesic regimens. Enhanced peripheral afferent activity is thought to initiate and maintain central sensitization at the spinal cord and higher levels of nociceptive sensory processing. Fos protein, the product of c-fos immediate early gene (IEG), has been used as a maker for neuronal activation in the central nervous system (CNS), which is rapidly activated after noxious stimuli to express Fos proteinin spinal dorsal horn neurons [17], [18]. To further clarify the analgesic effect of AGAP on pain induced by formalin, we investigate the effect of pre-treatment with AGAP on spinal Fos protein expression induced by formalin. The present results had shown that pre-treatment with AGAP inhibited the ipsilateral spinal Fos expression induced by formalin (Fig. 5A and B).

### AGAP potentiated the effects of the inhibitors of MAPKs on inflammatory pain

**Figure 6:**
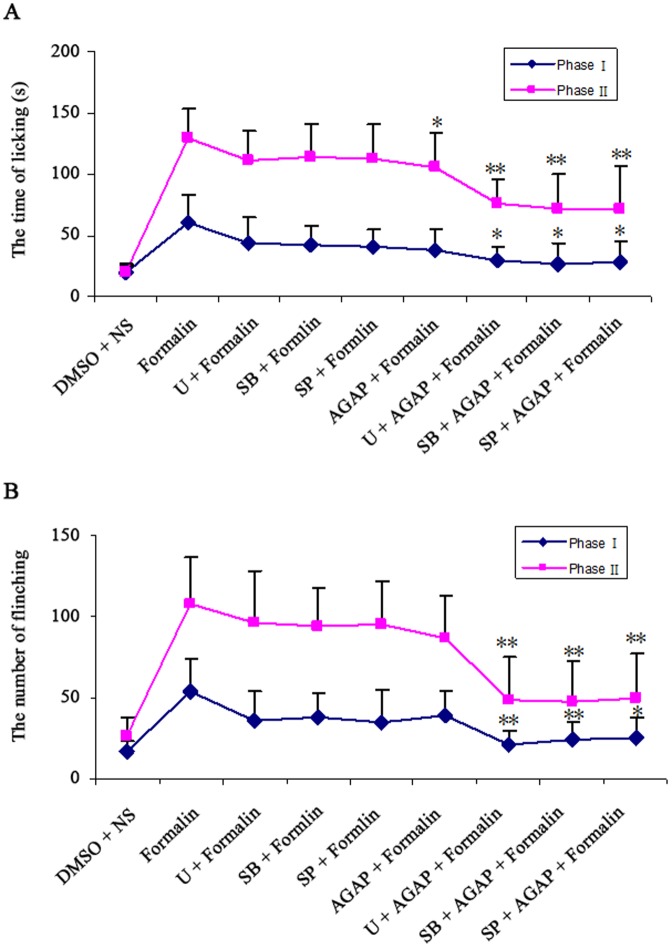
AGAP potentiated the effects of the inhibitors of MAPKs on inflammatory pain. The inhibitors of MAPKs (a p38 MAPK inhibitor SB203580, a MEK inhibitor U0126 or SP600125, a JNK MAPK inhibitor 0.1 µg) were injected at 30 min and AGAP (0.2 µg) was injected at 10 min before formalin injection, respectively. Spontaneous pain were recorded within 0∼5 min (Phase I) and 15∼30 min (Phase II) after formalin injection. Combinations the inhibitors with AGAP decreased the time of licking/biting paw (A) and the number of flinching paw (B). *P<0.05, **P<0.01 compared with formalin group, n = 8 mice in each group.

The above study has shown that pre-treatment with AGAP prevented the formalin-induced pain, which was associated with the inhibition of MAPKs. Therefore, AGAP will potentiate the effect of the inhibitors of MAPKs on pain behavior in theory. We found that pre-treatment with the lower doses inhibitors of MAPKs (a p38 MAPK inhibitor SB203580, a MEK inhibitor U0126 or SP600125, a JNK MAPK inhibitor 0.1 µg) at 30 min before formalin injection, had no effect on pain behavior. As we expected, treatment with AGAP at 20 min after the inhibitors injection, the total time of paw licking and number of paw elevation induced by formalin were significantly decreased (Fig. 6A and B).

## Discussion

This study demonstrated that (1) Pre-intraplantar injection AGAP prevents the inflammatory pain induced by formalin in a dose-dependent manner; (2) Formalin-induced inflammatory pain was accompanied with activation of peripheral and spinal MAPKs both in Phase I and II, and also was prevented by pre-treatment with AGAP; (3) Pre-treatment with AGAP inhibited the spinal Fos expression induced by formalin; (4) AGAP potentiated the effects of the inhibitors of MAPKs on inflammatory pain. These data demonstrate that pre-intraplantar injection AGAP prevents the inflammatory pain induced by formalin through a MAPKs-mediated mechanism in mice. These findings may have important implications for exploring the roles and mechanisms and for understanding the molecular basis of AGAP in analgesia. Therefore, our study suggested that AGAP would be useful in treatment of inflammation pain as an analgesia drug.

It is well known that intraplantar injection of formalin can induce biphasic spontaneous nociceptive responses. In general, the first-phase response is due to the high level of activity in the primary afferents induced by formalin, and the second phase was considered to be a tonic response resulting from the inflammation factor [14]–[17]. It has been demonstrated that BmK IT2, a kind of neruotoxic polypeptides, can suppress the biphasic nociceptive responses induced by formalin [6].The AGAP is an antitumor-analgesic peptide from the venom of Chinese scorpion Mesobuthus martensii Karsch [4]. Pharmacological assays showed that AGAP and its 12 mutants had effective analgesic activities compared with the negative control in the mouse-twisting reaction test, and the analgesic activities of 12 mutants were lower than that of AGAP [18]. Our percent study shows that pre-treatment with AGAP can dose-dependently prevent or suppress the inflammatory pain in formalin model.

Release of a broad range of sensitizers, including prostaniods, growth factors, cytokines, ATP, and TNF, from injury and/or inflammatory tissue, acting on the related receptors and signaling pathways on the peripheral terminal of nociceptors, induces a process of peripheral sensitization, which is an important neuronal mechanism underlying primary hyperalgesia at the site of injury or inflammation [19]. MAPKs, including p38, ERK, and JNK, are a family of serine/threonine protein kinases that transduce extracellular stimuli into intracellular posttranslational and transcriptional responses. It is well established that the MAPKs activation may be involved in the modulation of nociceptive information and peripheral and central sensitization produced by intense noxious stimuli through various routes [7]–[12]. Study has found that AGAP down-regulated the protein expression of pMAPK in *vitro* [13]. These studies suggested that MAPKs signaling may mediate the role of AGAP in preventing the inflammatory pain. We found that intraplantar injection of formalin induced inflammatory pain accompanied by activation of spinal MAPKs. Pre-treatment with AGAP prevents pain behavior and spinal and skin MAPKs expression induced by formalin. These findings indicated that MAPKs pathway was involved in the role of AGAP in preventing formalin-induced nociceptive response. Furthermore, AGAP could potentiate the effects of the inhibitors of MAPKs on the inflammatory pain. These findings indicated that MAPKs pathway was involved in the role of AGAP in preventing the nociceptive response and p-MAPKs-mediated mechanism contributes to both Phase I and II in formalin model.

Fos protein expression has been used as a maker for neuronal activation in the central nervous system and has been widely used as a tool in functional mapping of neuronal circuits in response to various defined stimuli. There is a positive correlation between the quantity of Fos protein expression and the degree of sensitization induced by nociceptive stimuli in spinal cord neurons. Long-term facilitation of c-fiber-evoked firing of wide dynamic range neurons in the spinal dorsal horn in response to conditioning stimulation of afferent fibers was accompanied with the frequency-dependent increase of c-fos-labeled cells in superficial, intermediate, and deep laminate of the dorsal horn on the stimulated side [20], [21]. In this study, we found that pre-intraplantar injection AGAP suppressed the increased expression of spinal Fos protein expression in formalin pain model. The decreased expression of spinal Fos protein further confirms the anti-nociceptive role of pre-treatment with AGAP.

## Conclusion

This study demonstrates that pre-intraplantar injection AGAP prevents the inflammatory pain induced by formalin through a MAPKs-mediated mechanism in mice. These findings may have important implications for exploring the roles and mechanisms and for understanding the molecular basis of AGAP in analgesia. Therefore, our study suggested that AGAP would be useful in treatment of inflammation pain as an analgesia drug.
